# Whole or Defatted Sesame Seeds (*Sesamum indicum* L.)? The Effect of Cold Pressing on Oil and Cake Quality

**DOI:** 10.3390/foods10092108

**Published:** 2021-09-06

**Authors:** Diana Melo, Manuel Álvarez-Ortí, Maria Antónia Nunes, Anabela S. G. Costa, Susana Machado, Rita C. Alves, José E. Pardo, Maria Beatriz P. P. Oliveira

**Affiliations:** 1 REQUIMTE/LAQV, Department of Chemical Sciences, Faculty of Pharmacy, University of Porto, Street of Jorge de Viterbo Ferreira 228, 4050-313 Porto, Portugal; melo_dian@hotmail.com (D.M.); antonianunes.maria@gmail.com (M.A.N.); anabelac020@gmail.com (A.S.G.C.); su_tche@hotmail.com (S.M.); rita.c.alves@gmail.com (R.C.A.); beatoliv@ff.up.pt (M.B.P.P.O.); 2 Higher Technical School of Agricultural and Forestry Engineering, University of Castilla-La Mancha, Campus Universitario, s/n, 02071 Albacete, Spain; manuel.alvarez@uclm.es

**Keywords:** nutritional analysis, amino acids profile, fatty acids profile, vitamin E profile, dietary fibre, antioxidants composition

## Abstract

Whole sesame seeds and sesame oil, which is obtained after cold pressing the seeds, are foodstuffs globally consumed due to their nutritional characteristics. The press cake that remains from the oil extraction process can be ground to form a defatted flour that can be incorporated into the human diet, contributing to the valorisation of this product. The nutritional comparison between the whole seeds and the press cake reveals the potential of this by-product to be incorporated in the formulation of diverse foodstuff, since it is richer than the seeds in proteins (30%) and fibre (25%) and still contains a proportion of oil (32%) with a fatty acid pattern characterized by the abundance of unsaturated fatty acids. The protein fraction of both the seeds and the cake shows a balanced composition regarding amino acid composition, with all the essential amino acids included. On the other hand, the oil obtained by cold pressing is shown as a high-quality oil, where the predominant fatty acids are oleic (42.66%) and linoleic (41.25%), which are essential fatty acids because they are not synthetised in the organism and must be obtained through the diet. In addition, it is rich in vitamin E, especially in γ-tocopherol, that was the main isomer found. Regarding these results, all products (sesame seeds, oil and press cake) are components suitable to be included in a healthy diet.

## 1. Introduction

Nowadays, consumers look for an adequate intake of nutrients and other bioactive compounds with beneficial health effects. They request functional foods with enhanced nutritional value and simultaneously natural ingredients. In this sense, seeds consumption is recommended due to several nutritional desirable properties [1,2]. Furthermore, with the rapid expansion of human population, there is a need for sustainable food production to ensure food security and the preservation of the environment [3]. However, today, about a third of food is wasted, including food industry residues that still contain high nutritional value and show a high potential for human consumption, since they are also source of some bioactive compounds. The valorisation of residues to turn them into by-products and incorporate them into the food chain follows the circular economy and eco-nutrition principles and ensures sustainability of the food chain [1,4].

Sesame (*Sesamum indicum* L.) is one of the oldest and most cultivated oilseeds worldwide. Their annual production in 2019 was higher than 6.5 million tons, led by Sudan, Myanmar and India, according to FAOSTAT data [5]. Sesame is essentially cultivated to produce oil, which has culinary uses such as the elaboration of bakery products, tahini sauce or for salads topping, among others [6,7,8,9], but also for cosmetics and dietary supplements [10]. The major importers of this oil are USA, Australia, Vietnam, Japan and the UK, but the major consumption comes from China and India [5].

Sesame oil has a pleasant aroma, colour and taste, characteristics highly influenced by edaphoclimatic conditions, plant cultivars and oil processing. These characteristics allow their use in salads, forcemeat and appetizers, or in margarine and cooking oils formulation. In relation to other vegetable oils, it has more unsaponifiable matter (≈2%), including phytosterols, triterpenic alcohols, tocopherols and lignans (mainly sesamin and sesamolin), providing a superior oxidative stability and several beneficial physiological effects [8].

The extraction of sesame oil leads to the generation of a defatted by-product—the sesame cake. This by-product from the sesame oil industry is commonly used as cattle feed or to make compost. However, this residue can be ground and converted into a flour ready to use with culinary purposes, with consequent added value for food industry. It contains protein with a balanced amino acid composition, dietary fibre and important bioactive compounds with antioxidant activity and health-promoting effects, such as lignans, mainly sesaminol triglucoside, sesamolinol diglucoside and sesaminol diglucoside [11,12].

The objective of this work was to assess the nutritional composition of sesame seeds available on the market and to verify their label compliance. Moreover, the chemical composition and antioxidant properties of cold-pressed sesame oil are also analysed, as well as the nutritional value of the remaining cake, with the aim of its valorisation as a food ingredient.

## 2. Materials and Methods

### 2.1. Sample Collection

White sesame seeds were acquired in a Spanish local market. The seeds were homogenized and divided in two batches, one for seed analysis and the other one for oil extraction. Before analysis, seeds were ground in a mill (GM Grindomix 200, Retsh, Haan, Germany). For oil extraction, 1 kg of seeds was subjected to cold pressing using a screw press Komet Oil Press CA59G (IBG Monforts Oekotec GmbH & Co. KG, Monchengladbach, Germany), at room temperature. The resulting oil was centrifuged to eliminate solid residues and was stored in dark glass bottles with minimum head space, under refrigeration conditions until analysis. The press cake was ground in a mill (GM Grindomix 200, Retsh, Germany) and sieved through a 1 mm sieve to homogenize the samples. The resulting flour was stored without oxygen in sealed plastic bags under refrigeration until analysis.

### 2.2. Sample Preparation and Analysis

Protein, fat, dietary fibre and ash of seeds and cake were analysed using standard methods [13] (Table 1). Moisture content was determined in an infrared moisture analyser (105 °C) (DBS-KERN & SOHN GmbH, Balingen, Germany). Ash content was quantified after incineration in a muffle furnace (500 °C, 24 h). Total protein content was determined by Kjeldahl procedure—acid digestion (H_2_SO_4_ 96%, 2 h) and distillation (titration with H_2_SO_4_ 0.5 M), using 6.25 as the nitrogen conversion factor [14]. Total fat content was determined by Soxhlet extraction (petroleum ether, 8 h). Total dietary fibre and insoluble fibre were analysed through enzymatic-gravimetric procedures. Soluble fibre and remaining carbohydrates were calculated by difference [14]. For energy estimation, the used values were fibre (2 kcal/g, 8 kJ/g), carbohydrates and protein (4 kcal/g, 17 kJ/g) and fat (9 kcal/g, 37 kJ/g) [15].

Seeds and cake free amino acids (AA) were extracted with water and total AA obtained by alkaline hydrolysis with KOH (for tryptophan) and acid hydrolysis with HCl (for the other AA). The extracts were analysed by HPLC-DAD-FLD (high-performance liquid chromatography-diode array detector-fluorescence light detector), using norvaline as internal standard, after ortho-phthalaldehyde/9-fluorenylmethyl chloroformate derivatization, in an Agilent ZORBAX Eclipse Plus C18 stationary phase column, according to Machado et al. [16].

For phytochemicals analysis, seeds and cake extraction was performed in constant agitation, using 25 mL of 80% methanol/water solution (40 °C, 1 h; MS-H-S10 magnetic stirrer, ChemLand, Poland), according to Costa et al. [17], with slight modifications. Oil phytochemicals were extracted with 80% methanol/water solution, according to Capannesi et al. [18]. The extracts were analysed by spectrophotometric methods (Microplate Synergy HT GENS5 Reader, BioTek Instruments, Winooski, Vermont, USA), namely, total phenolic compounds (TPC), according to Singleton and Rossi [19], with modifications proposed by Alves et al. [20], total flavonoids content (TFC) and 2,2-diphenyl-1-picrylhydrazyl radical (DPPH•) inhibition, following Costa et al. [21], and ferric reducing antioxidant power (FRAP), following Benzie and Strain [22], with modifications described by Costa et al. [21].

The lipid fraction of seeds/cake was extracted (for vitamin E and fatty acids profiles determination) as following: 150 mg of sample was weighted, 75 μL of BHT 0.1% (m/V) (antioxidant), 50 μL of tocol (0.1 mg/mL, internal standard) and 1 mL of absolute ethanol were added. The mixture was left in constant agitation (Multi Reax EU, Heidolph, Germany) for 30 min. Then, 2 mL of n-hexane were added, followed by another 30 min of agitation. In addition, 1 mL of NaCl 1% (m/V) was added. The mixture was centrifuged and the supernatant (n-hexane layer) was stored. Another 30 min extraction with 2 mL of n-hexane in agitation was performed, followed by centrifugation. Both supernatants were mixed and an appropriate amount of anhydrous sulphate was added. The extract was dried under nitrogen stream and resuspended in 1 mL of n-hexane for injection in the HPLC system, according to Alves et al. [23]. For vitamin E analysis, an HPLC-DAD-FLD equipment and a Supelco normal phase Supelcosil TM LC-SI column (75 mm × 3.0 mm, 3.0 μm) were used, as well as tocol as internal standard, according to Alves et al. [23].

For fatty acids (FA), transmethylation with KOH in methanol was performed according to ISO 12966-2:2017 [24], to obtain methyl esters. The profile was obtained by GC-FID (gas chromatography-flame ionization detector, Shimadzu, Tokyo, Japan), in a GC-2010 Plus gas chromatograph (Shimadzu, Tokyo, Japan) with an automatic sampler and a split/splitless auto injector (AOC-20i Shimadzu) operating with a 50:1 split ratio at 250 °C (injection), a Varian CP-Sil 88 silica capillary column (50.0 m × 0.25 mm inner diameter and 0.20 μm film thickness; Middelburg, The Netherlands) and a flame ionization detector (Shimadzu, Tokyo, Japan) at 270 °C. The analyses were performed using helium (3.0 mL/min) as the carrier gas and applying the following temperature programme: 120 °C held for 5 min, 2 °C/min to 160 °C, held for 2 min, and 2 °C/min to 220 °C, held for 10 min. The injection volume was 1.0 μL. The FA methyl esters were identified by comparison with FAME 37 standard mixture (Supelco, Bellefonte, PA, USA). Data were analysed based on relative peak areas, according to Nunes et al. [3].

Regarding the other parameters determined only in the oil, the following methodologies were used: oxidative stability by the Rancimat method (3 g of oil, 20 L/h air flow, at 120 °C; Rancimat apparatus, model 892, Metrohm), according to Szydłowska-Czerniak et al. [25]; chromatic coordinates (x, y), transparency (%), dominant wavelength (nm) and purity, according to NP-937:1987 [26] and Malheiro et al. [27]; peroxide value by titration with sodium thiosulfate, according to Kaleem et al. [28] and NP-904:1987 [29]; primary and secondary oxidation products, by spectrophotometry at 232 and 270 nm, respectively (Shimadzu UV Spectrophotometer UV-1800, Shimadzu, Tokyo, Japan), according to ISO 3656:2002 [30].

All determinations were obtained in triplicate.

Seeds label compliance was verified according to the tolerance ranges for carbohydrate/protein/fibre (<10%, ±2 g; 10–40%, ±20%; >40%, ±8 g, respectively) and fat (<10%, ±1.5 g; 10–40%, ±20%; >40%, ±8 g) [15,31].

For statistical analysis, an independent samples *t*-test was used to assess significant differences among samples at a 5% significance level (*p* < 0.05) (IBM SPSS Statistics, Windows v. 26, IBM Corp., Armonk, 241 NY, USA).

## 3. Results and Discussion

### 3.1. Nutritional Value of Seeds and Press Cake

The nutritional analysis of whole sesame seeds and press cake after oil extraction (Table 1) revealed differences in all parameters evaluated. Seeds had a higher caloric value than the cake, due to its higher fat content. Similar energy value (608 kcal/100 g) for sesame seeds has been reported Adebisi et al. [32]. The fat content of the seeds reached 53%, while, once subjected to oil extraction by cold pressing, the fat content of the press cake decreased to 32%. Pressure systems that allow cold extraction are generally the preferred method for oil extraction to obtain high quality virgin oils, although there is still an important oil fraction that remains in the press cake. This increases the nutritional value of the partially defatted flours, since the remaining oil provides its healthy characteristics, especially the high content in unsaturated fatty acids or vitamin E, to this by-product.

Regarding the other parameters (ash, protein, dietary fibre and carbohydrates) a different behaviour was displayed, with higher values found in the cake as a result of oil removal. Sesame seeds are known to be a source of several minerals, such as potassium (525.9 mg/100 g dry matter), phosphorus (516 mg/100 g dry matter), magnesium (349.9 mg/100 g dry matter), sodium (15.28 mg/100 g dry matter), iron (11.39 mg/100 g dry matter), zinc (8.87 mg/100 g dry matter), manganese (3.46 mg/100 g dry matter), copper (2.15 mg/100 g dry matter) and calcium (1.03% dry matter) [33]. The increase in the ash content of the press cake reveals that this sample is a bigger source of total minerals in comparison to the seeds.

Proteins are the nutrient with a higher increase in the cake (from 19 to 30% fw). Considering the high protein content in the samples, determining the amino acids (AA) profile may provide a better characterization to evaluate the protein quality. In addition, AA are involved in many physiological systems and metabolic functions. Thus, AA composition indicates the seeds and cake real protein contents and protein quality, which are important data when considering the nutritional value of a new ingredient [34]. A chromatographic approach (HPLC-DAD-FLD) allowed to determine total (after hydrolysis) and free AA profiles (Table 2).

The cake showed a significantly higher total AA content than seeds (305 and 199 mg/g, respectively). Regarding the total AA, the major AA identified (with more than 10 mg/g in seeds and 15 mg/g in cake, Table 2) were glutamic acid, arginine, aspartic acid, leucine, glycine, and serine, in descending order. Asparagine was not identified because, in hydrolysis conditions, it was totally converted into aspartic acid, as was the case of glutamine, that was converted in glutamic acid in acidic conditions [16]. In this study, hydroxyproline was detected in both samples, unlike other previous data [7,9,12].

The consumption of arginine has biological importance, as it is used by the nitric oxide synthase enzymes to produce nitric oxide in cells. In the central nervous system, it functions as a neurotransmitter, that may benefit the cerebral vascular system and the immune response, having a positive impact in learning and memory processes [35]. Arginine is also used in supplementation of athletes. Several studies showed that its consumption improved athletic performance and body composition, having a lowering effect in cardiovascular risk factors, that may be a result of increased levels of nitric oxide in serum [36].

On the other hand, glutamine, a non-essential AA, is the most abundant in the body and is involved in the intermediary metabolism and immune function. Immune cells use glutamine at a high rate, that is why dietary supplements of this AA (glutamine) are used by many elite athletes to re-establish the immune function after training sessions [37].

As regards free AA, both samples showed similar contents (2.0–2.7 mg/g fw). However, a different study [34] determined a higher total free AA (7.04 mg/g) in white sesame seeds. Nevertheless, the extraction and analysis conditions were different, which could have influenced the results. The major free AA identified (with more than 0.10 mg/g, Table 2) were asparagine, glutamic and aspartic acids followed by arginine, phenylalanine, and tryptophan.

All essential AA (phenylalanine, valine, threonine, tryptophan, methionine, leucine, isoleucine, lysine, and histidine) were present in seeds and cake, representing about 33% of the total AA content. Leucine was the major essential AA present in cake and seeds (20.7 and 13.6 mg/g, respectively) followed by phenylalanine and valine. Leucine plays a role in muscle growth. The branched-chain amino acids (BCAA), that include leucine, isoleucine and valine, are essential AA, which cannot be synthetised in the body and must be obtained through the diet, as they regulate human health, being the building blocks for protein synthesis. Several studies have also showed that free BCAA take part as regulators of protein and energy metabolism. In the brain, these AA are an active donor group for glutamate synthesis [38].

Overall, the protein of sesame seeds and cake showed a balanced AA composition, being dietary sources of all essential AA, including BCAA.

TDF (total dietary fibre) and insoluble fibre contents were higher in the cake by about 25% and 22% fw, respectively, in comparison to the seeds. These features meet consumers’ demand for protein- and fibre-rich foodstuffs. Overall, the cold pressing of seeds allowed to obtain a by-product (cake) with nutritional added value after extraction of the oil that can fit other consumer needs at food level. Dietary fibre is classified as soluble and insoluble, based on water solubility. Each type has different beneficial physiological effects. For example, soluble fibre has a positive impact on glycaemic response and plasma cholesterol levels, whereas insoluble fibre is mostly associated with increases in stool weight and reduction of the intestinal transit time [39].

A food product that contains both types of fibres presents enhanced physiological benefits. Both sesame seeds and press cake analysed samples showed a high-fibre content (Table 1), that provides several benefits to gastrointestinal microbiota stimulation, functioning as a prebiotic. A positive modulation of the microbiota impacts beneficially the host’s organism by promoting normal digestive function and defence against pathogens. In addition, soluble fibres form a viscous layer in the small intestine contributing to higher satiety, owing to lower macronutrients exposure to gut wall as nutrients reach further to the distal colon, resulting in appetite hormone changes, which may help in weight loss. Other beneficial effects are prolonged gastric emptying and lower rates of starch digestion, increased stool bulk and laxation, attenuation of post-prandial glycaemia and insulinaemia, decrease in total and low-density lipoprotein serum cholesterol levels, decreasing bile acids reabsorption and lowering blood pressure [40,41].

Hence, fibre is important to the prevention and treatment of diseases as obstipation, colon cancer, dyslipidaemia, and diabetes mellitus. High-consumed wheat and oat are examples of major sources of TDF in a healthy dietary pattern. These cereals can reach 13% (wheat) and 10% (oats) of TDF, of which 10% and 7% are insoluble and 2% and 4% are soluble fibres, respectively [42]. Results showed that cake presented 25% of TDF, reaching 22% of insoluble fibre and 3% of soluble fibre. The utilization of this by-product meets consumers’ request for gluten-free, natural, and high-fibre foods. At the same time, as it is obtained by a sustainable method with minimal processing, it fulfils the eco-nutrition principles.

Similar results have been reported, previously, regarding ash content (4.7%) and fat (52%) in sesame seeds, although slight differences have been found regarding the levels of protein (25.8%), TDF (19.3%), insoluble fibre (14%) and soluble fibre (5.4%) [33]. In comparison to the results of the present work, Joshi and colleagues, 2015, reported for sesame full fat seed flour lower contents of protein (14.01%), lipids (51.31%) and ash (3.48%), but higher contents of moisture (4.11%) and total carbohydrates (31.20% dry weight basis) [43]. As regards nutritional characteristics of the sesame press cake after oil extraction, lower contents of ash (6.1%), fat (30.3%), protein (23.7%) and fibre (10.8%) have been reported, but higher contents of moisture (5.71%) and carbohydrates (28.8%) [44]. These differences could be a result of different varieties and edaphoclimatic conditions to which the plants are subjected, which would therefore produce seeds with different contents in nutrients.

Regarding fat content, full sesame seeds contain 53.1% of fat. After oil cold extraction, the total fat content of the press cake was still high (31.8%); therefore, the oil from this product could be further re-extracted. Higher oil yields can be obtained using more drastic procedures (e.g., solvent extraction). The use of solvents for oil extraction increases the extraction efficiency, allowing to obtain higher oil yields, but may compromise oil and cake quality regarding their nutritional composition [45]. In addition, the use of high temperature in oil extraction by pressing may also increase the oil yield, promoting changes in seeds microstructure, as well as Maillard reactions, that lead to the liberation of aromas and darker colours. Nevertheless, to avoid losing quality (nutritional value and bioactive compounds) an optimization of the applied methods and conditions is advisable [6,8,9]. The press cake is an oil industry by-product that is generally considered as waste or used as cattle feed or for composting. However, it still contains a high nutritional value and has a great potential to be used as a food ingredient due to its nutritional features. Furthermore, plant-based protein can be an alternative to animal protein, meeting flexitarianism, vegetarianism or veganism food patterns. It may therefore be nutritional, social, and economically advantageous to incorporate it in new foodstuffs [11,12,46].

Fatty acids (FA) composition is also shown in Table 1. Seeds and cake had a similar profile regarding the SFA (saturated fatty acids, 17–16%), MUFA (monounsaturated fatty acids, 41%) and PUFA (polyunsaturated fatty acids, 42–43%), as expected. Moreover, the distribution of the FA is also comparable with the sesame seed oil. The most abundant FA were linoleic, oleic, and palmitic, whereas a low level of linolenic acid was detected.

Vitamin E is a fat-soluble antioxidant composed of different isomers of tocopherols and tocotrienols, which are present in vegetable oils and help prevent oxidation. Among these isomers, α-tocopherol possesses the highest biological activity, operating as a radical-chain breaking antioxidant in membranes, lipoproteins, and foods. On the other hand, γ-tocopherol is more powerful than α-tocopherol in low density lipid oxidation and in reducing platelet aggregation and intra-arterial thrombus formation [47]. In the vitamin E profile, three vitamers were identified in the sesame press cake and seeds, α-tocopherol, α-tocotrienol and γ-tocopherol, totalizing 226 and 432 mg/kg of sample fw in cake and seeds, respectively (Table 1). Nevertheless, Pathak et al. [47] reported a higher γ-tocopherol content in sesame seeds (800 mg/kg).

In addition, sesame seeds are also a source of other minor compounds with bioactive activities and health-promoting effects. Apart from vitamin E, they also contain thiamine (vitamin B1), phytosterols and lignans (sesamin, episesamin, sesamol, sesamolin, pinoresinol, sesaminol diglucoside and sesaminol triglucoside), among other phenolics. It has been reported that these antioxidants have antiproliferative activity, stimulate the function of hepatic FA oxidation enzymes, have lowering effects in cholesterol levels, blood pressure and serum lipids, decrease heart disease risk and prevent cancer development [48,49,50]. Phenolics have high antioxidant capacity, protect against oxidative damage, bind to biological polymers, chelate transient metal ions, neutralize free radicals and decompose peroxides. From a nutritional approach, obtaining food formulations rich in these compounds may decrease the use of antioxidant additives and/or improve the products nutritional profile, allowing to obtain healthier and functional foods [51]. Regarding the obtained results, sesame cake was significantly enriched in TPC, in comparison to seeds (153 and 74 mg GAE/100 g fw, respectively) (Table 1). These results may suggest that, although some phenolics are generally extracted with the oil, the main fraction remains in the press cake, since the values of total phenolics in the cake, once the oil has been removed, are higher in percentage than in the total seeds. Flavonoids are a sub-group of phenolics. Sesame cake was also significantly enriched in total flavonoids content (TFC), compared to raw seeds (74 and 67 mg ECE/100 g fw, respectively). Other previous studies have found higher values of TPC (up to 787 mg GAE/100 g) or TFC (up to 1355 mg catechin equivalents/100 g) in sesame seeds, although different extraction processes were used [33,44,52].

However, to evaluate the antioxidant properties of phenols and vitamin E in sesame seeds or cake, other analysis can be conducted. FRAP and DPPH• inhibition assays were the in vitro indirect methods for antioxidant activity determination of the samples. FRAP is based on the principle of metal reduction [51], whereas DPPH• inhibition assesses the antioxidant activity based on the capacity of some organic molecules to absorb radicals [51]. In the FRAP assay, there were no significant differences between sesame cake and seeds (8 and 7 mmol FSE/100 g fw, respectively). In the DPPH• inhibition assay, there were also no significant differences between sesame cake and seeds (78 and 86 mg TE/100 g fw, respectively). Thus, the press cake, after oil extraction, maintained the antioxidant ability of whole sesame seeds, suggesting that it can be used as an ingredient in food products to increase their health benefits.

The antioxidant activity of plant extracts is mainly attributed to the concentration of their phenolic compounds. The previous results suggest that there are constituents in the sesame seeds extracts able to scavenge free radicals via electron or hydrogen-donating mechanisms. Therefore, they could be capable of preventing the start of deleterious free radical-mediated chain reactions in vulnerable matrices, such as biological membranes. However, phytochemicals bioavailability and consequent bioactivity will suffer changes after processing and digestion processes of these products. As an example, the cooking process of sesame cake influenced antioxidant activity, namely FRAP and DPPH• inhibition results [53].

In another study, TPC and FRAP significantly increased with higher roasting temperatures, in comparison to unroasted seeds [54]. Overall, regarding the values of the sample’s antioxidant compounds and the potential reducing impact of processing on its content, it seems that it is not the most outstanding feature of sesame cake and other nutritional parameters can be exploited, such as the protein, TDF and minerals contents.

### 3.2. Vitamin E Profile of Seeds, Cake and Oil

Regarding the vitamin E profile of seeds, cake and oil (Table 1 and Table 3), it should be noticed that different extraction methods of vitamin E were used. For the oil, a direct method was used: 30 mg of cold pressed oil was weighed and mixed with 950 μL of n-hexane and 50 μL of tocol (0.1 mg/mL, internal standard); the solution was injected in the HPLC system. For the seeds/cake, the extraction process of vitamin E was different (as described in Section 2.2.) [23].

When a comparison of the vitamin E results of the oil and those of the seeds/cake was attempted, it was noticeable that the oil provided more γ-tocopherol (456 mg/kg of oil) than seeds and cake (431 and 222 mg/kg of sample, respectively). However, a different behaviour was displayed regarding the amounts of the other isomers, α -tocopherol (seeds, 118 mg/kg of seeds; cake, 57 mg/kg of cake; oil, 14 mg/kg of oil) and α-tocotrienol (seeds, 23 mg/kg of seeds; cake, 20 mg/kg of cake; oil, 14 mg/kg of oil), where the seeds/cake provided higher quantities. A possible explanation to this could be that as the extraction process for seeds/cake was longer (1 h 30 min) and another extracting solvent was used (absolute ethanol), it is possible that the plant cells (in seeds/cake) could further burst, allowing the release of higher amounts of some isomers of vitamin E (in this case, mainly α-tocopherol) bound to, e.g., membranes, since α-tocopherol is known to increase membrane rigidity; its concentration, together with that of the other membrane components, may be regulated to provide adequate fluidity for membrane function [55]. Moreover, a study reported that α-tocopherol can also be bound to tocopherol binding-proteins in plant cells, e.g., the tomato SlTBP protein, which targets chloroplasts and is able to bind α-tocopherol [56]. The method used for seeds/cake with constant agitation and with the use of absolute ethanol could have favoured the release of this vitamin E isomer into the final extract.

Another possible explanation could be the conversion of gamma-tocopherol into alpha-tocopherol in the seeds/cake. A review article highlighted the different biosynthetic pathways of vitamin E in plants. It was reported that, unlike leaves, the tocochromanol composition of wild-type seeds is much more variable. The seeds of some plants accumulate mostly γ-tocochromanols and very little or no α-tocochromanols. While other seeds, as a result of the activity of the gene γ-TMT/VTE4, are able to quantitatively convert almost all of the γ-tocochromanols into α-tocochromanols. In the biosynthetic pathways, tocochromanols are the precursors of tocopherols, tocotrienols and tocomonoenols [57].

### 3.3. Fatty Acids Profile of Seeds, Cake and Oil

**Figure 1:**
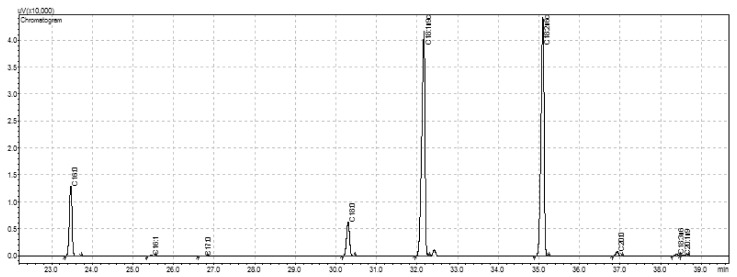
A typical GC-FID (gas chromatography coupled to flame ionization detector) chromatogram obtained for sesame seeds fatty acids profile (C16:0, palmitic; C16:1, palmitoleic; C17:0, margaric; C18:0, stearic; C18:1n9c, oleic; C18:2n6c, linoleic; C20:0, arachidic; C18:3n3, linolenic; C20:1n9, eicosenoic acids), the figure is presented with zoom for better view of the peaks of the fatty acids methyl esters, the analytical run was carried out for 65 min.

Sesame oil is composed of triglycerides, diglycerides, free FA, polar lipids and monoglycerides and cold pressing the seeds preserves its nutritional value [6]. Among seeds, cake and oil, the FA profile was similar (Figure 1). Palmitic and stearic acids contents varied between 9 and 10% and 5 and 6%, respectively. Palmitoleic, linolenic, arachidic and eicosenoic acids contents were <1%. The major FA in the seeds and cake was linoleic acid (42%), followed by oleic acid (41%). Cake presented more PUFA content. The opposite was found for the oil (Table 3), where the major FA was oleic acid (43%, MUFA), followed by linoleic acid (41%). These results are consistent with data from literature for seeds, cake and oil [8,58,59]. Thus, all three products (seeds, oil and press cake) may be considered as important dietary sources of oleic and linoleic acids.

The European diet is rich in linoleic acid and SFA [2]. Sesame n6/n3 ratio was high, due to low linolenic acid content, which does not counterbalance with this type of diet. Nevertheless, the SFA content was low (≈16%).

The consumption of sesame seed oil, alone or contained in the seeds or cake, may help to increase the intake of linoleic acid, an essential FA that cannot be synthesized by the human body. In humans, linoleic acid (PUFA) is converted by a series of desaturation and elongation reactions to its respective longer chains, as arachidonic acid, with several positive outcomes related to human health if consumed in moderate amounts. It was reported that 1–2% of linoleic acid is converted in other n6 PUFAs, of which only 0.5% appears as arachidonic acid [60].

A second extraction can be performed in sesame press cake using chemical solvents to increase the oil yield. However, the products obtained with this method cannot be used as a natural and chemical-free food ingredient [8]. Thus, the use of cold pressing cake is encouraged for the development of new food products, since its nutritional profile is advantageous—particularly the FA profile, where oleic acid outstands. The search for new sources of vegetable oils includes the use of residues that can be subjected to extraction to obtain oils rich in unsaturated fatty acids. This is the case of the prickly pear fruit waste, which contains seeds whose oil has also been proposed as an alternative source of oleic acid [61].

### 3.4. Characterization of Cold Pressed Sesame Oil

Chemical analysis of cold-pressed sesame oil (Table 3) revealed a low induction time when oxidative stability was evaluated (6 h). Lower oxidative stability values are generally related with a high content in PUFA, e.g., chia oil (higher PUFA content of 80%, lower oxidative stability of 2 h) [2]. Sesame oil presented an SFA content of 16% and a high content of MUFA (43%), resulting in a more stable oil. Similar values were obtained in other studies [62], but, when the oxidative stability was measured in oils extracted from coated seeds, the induction time was higher than the obtained in the oil extracted from dehulled seeds [33], such as those studied in this work.

The presence of endogenous antioxidants may play a role in the stability to oxidation. In this sense, phenolics stabilize edible oils, minimize rancidity, reduce toxic oxidation products formation, contribute to maintaining nutritional quality and extend the shelf-life [63]. In addition, tocopherols inhibit oil oxidation, act as free radicals’ biological scavengers and have nutritional importance as a source of vitamin E [59]. Although the main fraction of phenolics remain in the press cake, these compounds were also observed in the oil. Thus, TPC and TFC found in the oil were 13 mg GAE/100 g and 20 mg ECE/100 g, respectively. In this sense, the phenol extraction method can lead to different values [33,45].

In addition, the presence of vitamin E is a remarkable characteristic of edible vegetable oils. Moreover, sesame oil contains several liposoluble antioxidants (e.g., sesamin, sesamolin), which stimulate vitamin E activity against lipid peroxidation [64]. Sesamin is a dietary enterolignan precursor, metabolised to enterolactone by intestinal bacteria; its consumption may benefit hormonal metabolism, angiogenesis, anti-oxidation and activation of oestrogen receptors [65]. The total content of vitamin E found in the cold press sesame oil was 483.95 mg/kg (Table 3), where γ-tocopherol was the principal isomer. This result is consistent with previous studies [66], although, in some cases, the reported value for γ-tocopherol is slightly higher [47].

Unlike the present results, another work identified the four tocopherols, α-, β-, γ- and δ-tocopherol, although at a very low level [59].

PUFA oxidation, in sesame oil mainly linoleic acid (41%), can be followed by an increase of the values of the extinction coefficients K_232_ and K_270_, measured by UV absorption. Lipids with dienes or polyenes suffer a shift in their double-bond position during oxidation due to isomerization and formation of conjugates. Conjugated dienes exhibit an intense absorption at 232 nm and conjugated trienes at 270 nm. Lipid peroxidation can be accelerated by heating and bad storage conditions, leading to changes in UV absorption [67].

According to the results observed in the cold-pressed sesame oil, no products of primary (K_232_) and secondary oxidation (K_270_) were formed. These results, together with the low values of the peroxide value (2.6 meqO_2_/kg), show the high quality of the oils extracted by mechanical methods at low temperatures, which, unlike solvent extraction, lead to the elaboration of virgin oils ready for consumption. However, oil extraction conditions may originate oxidation processes that originate higher values of these parameters [10,68].

Regarding antioxidant activity, the FRAP value for sesame oil was 216 μmol FSE/100 g and DPPH• inhibition assay; sesame oil showed a value of 8 mg TE/100 g. A previous study evaluated sesame oil antioxidant stability by FRAP analysis, reporting a decrease from 803 to 763 μmol/L after one month [69].

### 3.5. Seed’s Label Compliance

Since consumers are currently more informed and want to know the correct nutritional composition of what they eat, seeds label compliance was verified (Table 4). According to the guidance document, the amount of a nutrient in a product may vary in comparison to the declared label value due to numerous reasons: the reference values used to develop the nutritional information, the analysis accuracy, raw materials variation, processing effect, nutrient stability, storage conditions and time. In this work, carbohydrates and dietary fibre contents were outside the tolerance ranges. A value can be outside the tolerance range as a result of many factors: the different type of nutrient in question; the extent and nature of the value’s deviation; the natural high variation of the nutrient in the food matrix, due to, e.g., seasonality; the different degradation rate of the nutrient in the food matrix; the analytical variability of the nutrient’s value in the food matrix; low homogeneity of a food product, leading to a high variation of the nutrient’s content in the food product; compliance of the majority of samples from the lot with the tolerance range; validity of the manufacturer’s process for establishing the declared nutrient value; how the self-monitoring method of the company is implemented [31]. The aspects mentioned above could explain the differences obtained between the label and the present results. Nevertheless, a review of the declared information in the label is recommended.

## 4. Conclusions

Cold-pressed sesame oil is a source of linoleic and oleic acids and γ-tocopherol. The press cake obtained after oil extraction showed a great potential for formulation of foodstuffs as a source of protein, total minerals and dietary fibre. It can be incorporated by the food industry and valorised for consumption, meeting consumers’ request for gluten-free and natural products, obtained in a sustainable, minimal processed way, without the addition of preservatives and stabilizers. Plant protein can be an alternative to animal protein, meeting current plant-based food patterns.

The seeds comprise the nutritional characteristics mentioned above. In the current food market, the search for sesame seed products is increasing, which is likely to be a reality in the future.

These products presented phenolics which can provide health benefits. Nevertheless, there is a need to assess their content and bioavailability after processing/digestion and validate biological effects. Overall, the nutritional features conveyed by the consumption of these products would be noticeable when included in a varied food pattern and active lifestyle.

## Figures and Tables

**Table 1:** Chemical analysis of sesame seeds and cake.

Parameter	Method of Analysis	Fresh Weight	
		Seeds	Cake
Moisture (%)	Infrared moisture analyser	2.7 ± 0.1 ^a^	3.7 ± 0.2 ^b^
Ash (%)	Incineration (AOAC 920.153)	5.1 ± 0.0 ^a^	7.1 ± 0.0 ^b^
Total fat (%)	Soxhlet method (AOAC 991.36)	53.1 ± 0.0 ^b^	31.8 ± 0.4 ^a^
Fatty acids profile (relative%)			
C16:0	GC-FID	10.38 ± 0.20 ^b^	9.23 ± 0.01 ^a^
C16:1	GC-FID	0.13 ± 0.00 ^a^	0.12 ± 0.00 ^a^
C17:0	GC-FID	0.06 ± 0.01 ^a^	0.06 ± 0.00 ^a^
C18:0	GC-FID	5.63 ± 0.07 ^a^	6.13 ± 0.02 ^b^
C18:1n9c	GC-FID	41.03 ± 0.20 ^a^	40.92 ± 0.01 ^a^
C18:2n6c	GC-FID	41.67 ± 0.11 ^a^	42.42 ± 0.02 ^b^
C18:3n3	GC-FID	0.32 ± 0.01 ^a^	0.33 ± 0.00 ^a^
C20:0	GC-FID	0.60 ± 0.02 ^a^	0.61 ± 0.00 ^a^
C20:1n9	GC-FID	0.18 ± 0.01 ^a^	0.17 ± 0.00 ^a^
∑ SFA	GC-FID	16.67 ^b^	16.03 ^a^
∑ MUFA	GC-FID	41.34 ^a^	41.21 ^a^
∑ PUFA	GC-FID	41.99 ^a^	42.76 ^b^
18:2n6/18:3n3	GC-FID	130.09	127.02
18:1n9/18:2n6	GC-FID	1.02	1.04
Total vitamin E (mg/kg)	HPLC-DAD-FLD	432.03 ^b^	225.75 ^a^
α-Tocopherol (mg/kg)	HPLC-DAD-FLD	118.43 ± 4.82 ^b^	57.16 ± 1.36^á^
α-Tocotrienol (mg/kg)	HPLC-DAD-FLD	23.23 ± 2.29 ^b^	19.77 ± 1.51 ^a^
γ-Tocopherol (mg/kg)	HPLC-DAD-FLD	290.37 ± 12.81 ^b^	146.16 ± 6.29 ^a^
Protein (%)	Kjeldahl method (AOAC 928.08) × 6.25 [14]	18.9 ± 0.0 ^a^	29.6 ± 0.0 ^b^
Total amino acids (mg/g)	HPLC-DAD-FLD	199.44 ^a^	305.42 ^b^
Free amino acids (mg/g)	HPLC-DAD-FLD	2.02 ^a^	2.67 ^b^
Total Dietary Fibre (%)	Enzymatic-gravimetric (AOAC 985.29)	19.9 ± 0.0 ^a^	25.0 ± 0.6 ^b^
Insoluble Fibre (%)	Enzymatic-gravimetric (AOAC 991.42)	18.2 ± 0.0 ^a^	21.7 ± 0.0 ^b^
Soluble Fibre (%)	Calculated by difference [14]	1.7 ± 0.0 ^a^	3.3 ± 0.4 ^b^
Remaining Carbohydrates (%)	Calculated by difference [14]	0.3 ± 0.1 ^a^	2.9 ± 0.3 ^b^
Energy value (kJ/100 g)	Calculated [15]	2452	1927
Energy value (kcal/100 g)	Calculated [15]	595	466
Phytochemicals			
TPC (mg GAE/100 g)	Spectrophotometric (765 nm)	73.6 ± 5.7 ^a^	153.2 ± 6.1 ^b^
TFC (mg ECE/100 g)	Spectrophotometric (510 nm)	66.7 ± 5.2 ^a^	74.0 ± 3.8 ^b^
Antioxidant activity			
FRAP (mmol FSE/100 g)	Spectrophotometric (595 nm)	6.8 ± 0.5 ^a^	8.3 ± 0.3 ^b^
DPPH• (mg TE/100 g)	Spectrophotometric (Kinetics reaction, 525 nm)	86.4 ± 4.4 ^a^	78.2 ± 12.1 ^a^

Values presented as mean ± standard deviation (*n* = 3). Different letters denote significant differences by independent samples *t*-test (*p* < 0.05). Results of fatty acids in relative percentage (%). C16:0, palmitic; C16:1, palmitoleic; C17:0, margaric; C18:0, stearic; C18:1n9c, oleic; C18:2n6c, linoleic; C18:3n3, linolenic; C20:0, arachidic; C20:1n9, eicosenoic acids; SFA, saturated; MUFA, monounsaturated; PUFA, polyunsaturated fatty acids; TPC, total phenolic compounds; GAE, gallic acid equivalents; TFC, total flavonoids content; ECE, epicatechin equivalents; FRAP, ferric reduction antioxidant power; FSE, ferrous sulphate equivalents; DPPH•, 2,2-diphenyl-1-picrylhydrazyl radical; TE, trolox equivalents; GC-FID, gas chromatography-flame ionization detector; HPLC-DAD-FLD, high-performance liquid chromatography-diode array detector-fluorescence light detector.

**Table 2:** Amino acids profile of sesame seeds and cake.

	Total Amino Acids (mg/g of Sample fw)		Free Amino Acids (mg/g of Sample fw)	
	Seeds	Cake	Seeds	Cake
Aspartic acid	16.77 ± 0.68 ^a^	26.20 ± 0.73 ^b^	0.25 ± 0.04 ^A^	0.33 ± 0.04 ^B^
Glutamic acid	39.90 ± 1.51 ^a^	61.93 ± 1.80 ^b^	0.33 ± 0.04 ^A^	0.45 ± 0.04 ^B^
Asparagine	ND	ND	0.46 ± 0.02 ^A^	0.67 ± 0.03 ^B^
Serine	10.05 ± 0.34 ^a^	15.20 ± 0.48 ^b^	0.07 ± 0.00 ^A^	0.09 ± 0.00 ^B^
Glutamine	1.06 ± 0.02 ^a^	1.93 ± 0.04 ^b^	0.03 ± 0.00 ^A^	0.03 ± 0.00 ^A^
* Histidine	6.93 ± 0.40 ^a^	10.45 ± 0.12 ^b^	0.04 ± 0.00 ^A^	0.06 ± 0.00 ^B^
Glycine	11.21 ± 0.43 ^a^	17.41 ± 0.53 ^b^	0.02 ± 0.00 ^A^	0.03 ± 0.00 ^B^
* Threonine	7.38 ± 0.33 ^a^	11.31 ± 0.40 ^b^	0.05 ± 0.00 ^A^	0.05 ± 0.00 ^B^
Arginine	31.71 ± 1.38 ^a^	48.31 ± 1.43 ^b^	0.15 ± 0.00 ^A^	0.19 ± 0.01 ^B^
Alanine	9.57 ± 0.34 ^a^	14.60 ± 0.44 ^b^	0.09 ± 0.01 ^A^	0.11 ± 0.00 ^B^
Tyrosine	5.98 ± 0.36 ^a^	9.07 ± 0.21 ^b^	0.03 ± 0.00 ^A^	0.05 ± 0.00 ^B^
* Valine	8.40 ± 0.35 ^a^	13.09 ± 0.33 ^b^	0.04 ± 0.00 ^A^	0.06 ± 0.01 ^B^
* Methionine	5.06 ± 0.31 ^a^	6.97 ± 0.30 ^b^	0.03 ± 0.00 ^A^	0.03 ± 0.00 ^B^
* Tryptophan	1.89 ± 0.07 ^a^	2.19 ± 0.05 ^b^	0.12 ± 0.00 ^A^	0.15 ± 0.01 ^B^
* Phenylalanine	9.27 ± 0.36 ^a^	13.87 ± 0.53 ^b^	0.14 ± 0.00 ^A^	0.18 ± 0.02 ^B^
* Isoleucine	7.08 ± 0.29 ^a^	11.12 ± 0.24 ^b^	0.03 ± 0.00 ^A^	0.05 ± 0.00 ^B^
* Leucine	13.63 ± 0.55 ^a^	20.70 ± 0.57 ^b^	0.04 ± 0.00 ^A^	0.05 ± 0.00 ^B^
* Lysine	6.58 ± 0.49 ^a^	10.32 ± 0.30 ^b^	0.04 ± 0.00 ^A^	0.05 ± 0.00 ^B^
Hydroxyproline	0.50 ± 0.03 ^a^	0.73 ± 0.02 ^b^	0.01 ± 0.00 ^A^	0.01 ± 0.00 ^B^
Proline	6.58 ± 0.12 ^a^	10.04 ± 0.31 ^b^	0.03 ± 0.00 ^A^	0.05 ± 0.00 ^B^
Total	199.53 ^a^	305.45 ^b^	2.02 ^A^	2.67 ^B^

* Essential amino acids. Values presented as mean ± standard deviation (*n* = 3). Different letters denote significant differences by independent samples *t*-test (*p* < 0.05). ND, not detected.

**Table 3:** Chemical analysis of cold pressed sesame oil.

Parameter	Method of Analysis	Oil
Fatty acids profile (relative%)		
C16:0	GC-FID	9.74 ± 0.06
C16:1	GC-FID	0.10 ± 0.03
C18:0	GC-FID	5.41 ± 0.09
C18:1n9c	GC-FID	42.66 ± 0.35
C18:2n6c	GC-FID	41.25 ± 0.22
C18:3n3	GC-FID	0.20 ± 0.03
C20:0	GC-FID	0.54 ± 0.04
C20:1n9	GC-FID	0.11 ± 0.02
∑ SFA	GC-FID	15.68
∑ MUFA	GC-FID	42.87
∑ PUFA	GC-FID	41.45
18:2n6/18:3n3	GC-FID	203.94
18:1n9/18:2n6	GC-FID	1.03
Total vitamin E (mg/kg)	HPLC-DAD-FLD	483.95 ± 7.11
α-Tocopherol (mg/kg)	HPLC-DAD-FLD	13.78 ± 0.85
α-Tocotrienol (mg/kg)	HPLC-DAD-FLD	14.04 ± 0.29
γ-Tocopherol (mg/kg)	HPLC-DAD-FLD	456.13 ± 6.05
Phytochemicals		
TPC (mg GAE/100 g)	Spectrophotometric (765 nm)	13.4 ± 1.3
TFC (mg ECE/100 g)	Spectrophotometric (510 nm)	20.3 ± 1.7
Antioxidant activity		
FRAP (μmol FSE/100 g)	Spectrophotometric (595 nm)	215.5 ± 6.7
DPPH• (mg TE/100 g)	Spectrophotometric (Kinetics reaction, 525 nm)	7.9 ± 0.9
Oxidative stability		
Induction time (h)	Rancimat method	5.8 ± 0.0
Colour		
Chromatic coordinates (x, y)	Spectrophotometric (445, 495, 560, 595, 625 nm)	(0.3641, 0.3647)
Transparency (%)	Spectrophotometric (445, 495, 560, 595, 625 nm)	73.2
Dominant wavelength (nm)	Spectrophotometric (445, 495, 560, 595, 625 nm)	579.3
Purity	Spectrophotometric (445, 495, 560, 595, 625 nm)	27.4
Peroxide value (meqO_2_/kg)	Titration	2.6 ± 0.3
K_232 nm_	Spectrophotometric (232 nm)	0.03
K_270 nm_	Spectrophotometric (270 nm)	0.01

Values presented as mean ± standard deviation (*n* = 3), except for colour and oxidation products parameters (*n* = 1). Results of fatty acids in relative percentage (%). C16:0, palmitic; C16:1, palmitoleic; C18:0, stearic; C18:1n9c, oleic; C18:2n6c, linoleic; C18:3n3, linolenic; C20:0, arachidic; C20:1n9, eicosenoic acids; SFA, saturated; MUFA, monounsaturated; PUFA, polyunsaturated fatty acids; TPC, total phenolic compounds; GAE, gallic acid equivalents; TFC, total flavonoids content; ECE, epicatechin equivalents; FRAP, ferric reduction antioxidant power; FSE, ferrous sulphate equivalents; DPPH•, 2,2-diphenyl-1-picrylhydrazyl radical; TE, trolox equivalents; K, extinction coefficient; GC-FID, gas chromatography-flame ionization detector; HPLC-DAD-FLD, high-performance liquid chromatography-diode array detector-fluorescence light detector; ND, not detected.

**Table 4:** Sesame seeds label compliance according to the guidance document for competent authorities for the control of compliance with EU legislation on Regulation (EU) No. 1169/2011 of the European Parliament and of the Council of 25 October 2011 on the provision of food information to consumers.

Parameter	Label	Tolerance	Lower Tolerance	Upper Tolerance	Obtained Result	Comparison with the Tolerance Range
Energy value (kJ/100 g)	2654	-	-	-	2518	-
Energy value (kcal/100 g)	632	-	-	-	611	-
Fat (g/100 g)	55.9	±8 g	47.9	63.9	54.6	Within
Carbohydrates (g/100 g)	13.3	±20%	10.6	16.0	0.3	Outside
Dietary fibre (g/100 g)	7.6	±2 g	5.6	9.6	20.4	Outside
Protein (g/100 g)	17.6	±20%	14.1	21.1	19.4	Within

Values in dry weight.

## Data Availability

Not applicable.
